# The glycaemic outcomes of Cinnamon, a review of the experimental evidence and clinical trials

**DOI:** 10.1186/s12937-015-0098-9

**Published:** 2015-10-16

**Authors:** Arjuna B. Medagama

**Affiliations:** Department of Medicine, Senior Lecturer in Medicine, University of Peradeniya, Peradeniya, Sri Lanka

**Keywords:** Type 2 diabetes mellitus, Impaired fasting glucose, Cinnamon, Glucose transporter type 4 (GLUT4), Coumarins, Glycaemic control

## Abstract

**Introduction:**

Cinnamon is currently marketed as a remedy for obesity, glucose intolerance, diabetes mellitus and dyslipidaemia. Integrative medicine is a new concept that combines conventional treatment with evidence-based complementary therapies.

**Aim:**

The aim of this review is to critically evaluate the experimental evidence available for cinnamon in improving glycaemic targets in animal models and humans.

**Results:**

Insulin receptor auto-phosphorlylation and de-phosphorylation, glucose transporter 4 (GLUT-4 ) receptor synthesis and translocation, modulation of hepatic glucose metabolism through changes in Pyruvate kinase (PK) and Phosphenol Pyruvate Carboxikinase (PEPCK), altering the expression of PPAR (γ) and inhibition of intestinal glucosidases are some of the mechanisms responsible for improving glycaemic control with cinnamon therapy.

We reviewed 8 clinical trials that used *Cinnamomum cassia* in aqueous or powder form in doses ranging from 500 mg to 6 g per day for a duration lasting from 40 days to 4 months as well as 2 clinical trials that used cinnamon on treatment naïve patients with pre-diabetes. An improvement in glycaemic control was seen in patients who received Cinnamon as the sole therapy for diabetes, those with pre-diabetes (IFG or IGT) and in those with high pre-treatment HbA1c. In animal models, cinnamon reduced fasting and postprandial plasma glucose and HbA1c.

**Conclusion:**

Cinnamon has the potential to be a useful add-on therapy in the discipline of integrative medicine in managing type 2 diabetes. At present the evidence is inconclusive and long-term trials aiming to establish the efficacy and safety of cinnamon is needed. However, high coumarin content of *Cinnamomum cassia* is a concern, but *Cinnamomum zeylanicum* with its low coumarin content would be a safer alternate.

## Background

### Cinnamon

*Cinnamon*, which is derived from a Greek word that means sweet wood, comes from the inner bark of tropical evergreen cinnamon trees [1]. *Cinnamomum* (cinnamon) is a genus of the Lauraceae family, many of whose members are used as spices [2]. It is one of the most widely used flavouring agents used in the food and beverage industry worldwide and well recognized for its medicinal properties since antiquity. Traditional Ayurvedic medicine has used cinnamon extracts for ailments such as arthritis, diarrhoea and menstrual irregularities [3].

To date, about 250 species of cinnamon has been identified, 4 of which are used to obtain the spice cinnamon. True or Ceylon Cinnamon (*Cinnamomum verum*) (syn *C. zeylanicum*) is a small evergreen tree native to Sri Lanka. Chinese cassia cinnamon (*Cinnamomum cassia*) is the other most widely available species [4].

Preparation of cinnamon involves stripping of the outer bark of the tree and letting the inner bark to dry and curl up into its customary cinnamon quills. Cinnamon is available in either its whole quill form (Cinnamon sticks) or as ground powder in the market [5].

At present Cinnamon is sold as both a preventative and therapeutic supplement for many ailments including, metabolic syndrome, insulin resistance, type 2 diabetes, hyperlipidaemia and arthritis [5].

Cinnamon is known to have anti-diabetic properties, in addition to which, it is also perceived to have anti-oxidant, anti-inflammatory and anti-bacterial properties [6, 7].

### Burden of diabetes and use of complementary medicines

Increasing prevalence of diabetes mellitus is a major cause of morbidity and mortality worldwide. The WHO estimates a prevalence of 347 million people with diabetes worldwide in 2015 [8]. Diabetes mellitus is an illness, where a wide array of complementary and alternative medicines (CAMs) has been used with varying success. Around 2-3.6 million people in the United States rely on complementary and alternative medicines for the treatment of their diabetes mellitus [9].

Studies have been consistent in showing that diabetic patient adherence to current conventional treatment protocols are poor [10]. Diabetic patients are 1.6 times more likely than non-diabetics to use a CAM for a host of reasons [11].

Complex treatment regimes, hypoglycaemia, patient beliefs and side effects of medications have been compelling reasons that limited patient compliance with conventional treatment [9].

The worldwide trend for the use of CAMs in diabetes has increased with an overall prevalence ranging between 30-57 % [12].

Recent estimates show that over 80 % of people living in developing countries depend on CAM for treatment of health conditions [9]. In the United States an increase of 380 % is seen in the use of herbal remedies [13]. The economic burden of CAMs is substantial, with the UK population in 2001 having spent an estimated 340 million pounds on CAMs [14]. More recently in 2013, Herman estimates the cost of Complementary and Integrative Medicine (CIM) in the US to be 34 billion dollars [15].

Integrative medicine is a new discipline of medicine that combines conventional medicine with evidence-based complementary medicines. Patients with diabetes are increasingly eager to be part of their disease management and keen to try out integrative strategies that involve lifestyle changes and self management [16].

Therefore it is timely to review the evidence that supports its use at both molecular and clinical levels.

### Pathogenesis of Type 2 diabetes and insulin resistance

Type 2 diabetes is a complex metabolic disorder of insulin sensitivity and action on peripheral tissues like skeletal muscle and adipose tissue and of impaired insulin secretion.

Insulin signaling involves a cascade of events initiated by insulin binding to its cell surface receptor. The receptor consists of 2 α and 2 β subunits that is disulfide linked into a hetero-tetrameric complex. Insulin binds to the extracellular α subunits, activating the intra cellular tyrosine kinase domain of the β subunit. This is followed by receptor autophosphorylation, and activation of receptor tyrosine kinases, which result in tyrosine phosphorylation of insulin receptor substrates (IRSs) including IRS1, IRS2, IRS3, IRS4, Gab1, and Shc [17].

The β subunit is known to undergo serine/threonine phosphorylation, which decreases its ability to autophosphorylate to initiate the phosphorylation of the IRSs in insulin resistant animal models and humans. The activation of AMP activated protein kinase (AMPK) induces the translocation of glucose transporter 4 (GLUT4) to the plasma membrane [18], and several studies have demonstrated that AMPK and its signaling pathway are potential molecular targets in the development of drugs for the treatment of type 2 diabetes and obesity [19].

IRSs also leads to translocation of insulin mediated GLUT-4 from intracellular vesicles to the plasma membrane, facilitating glucose entry. In the same process, the insulin receptor is dephosphorylated and inactivated. This internalization and the dephosphorylation of the insulin receptor are done by the protein tyrosine phosphatases. Therefore the physiological action of insulin is a delicate balance between phosphorylation and dephsophorylation. Phosphatidylinositol 3-kinase (PI3K) is a key component of the insulin-signaling cascade, essential for the metabolic effects of insulin on glucose transport and GLUT4 translocation [17].

Derangement of insulin signaling pathways such as the impaired activation of the phosphatidylinositol 3-kinase and downstream signaling is thought to be responsible for the onset of insulin resistance and diabetes.

### Chemical composition, active constituents and metabolism

A plant is made up of many thousands of bioactive compounds and isolation of such compounds can be tedious and time consuming.

Jayaprakasha et al in 2011, using hydro-distillation techniques attempted to isolate the volatile oils of Cinnamon contained in the leaves and the bark. The major compounds were found to be Eugenol, cinnamaldehyde, copane, cinnamyl acetate and camphor, along with minor constituents [4]. Previous investigators had isolated Cinnamaldehyde, cinnamyl acetate and camphor from the bark. The active compound of Cinnamon with hypoglycaemic activity is a contentious issue [20]. To date, multiple compounds with hypoglycaemic properties have been isolated and used in both animal and clinical trials. A few such compounds are briefly discussed.

Babu et al used cinnamaldehyde extracted from *Cinnamomum zeylanicum* to demonstrate a significant reduction of plasma glucose and HbA1c levels in streptozotcin induced diabetic rats [21]. This study was carried out to isolate and identify the putative anti-diabetic compounds based on bioassay-guided fractionation. Cinnamaldehyde thus isolated was orally fed to diabetic rats, which caused significant reduction of plasma glucose and increased insulin levels. The authors concluded that cinnamaldehyde is responsible for increasing insulin secretion from pancreatic β cells and thus ameliorating glucose levels [21].

The stem of *cinnamomum cassia* (Cinnamoni ramulus) has been used in rats to determine its pharmacokinetic properties. The essential oil of cinnamoni ramulus is made up of a large number of aromatic compounds, which include cinnamaldehyde, cinnamic acid and 2-methoxy cinnamic acid. Bin Ji and colleagues demonstrated that following the oral administration of 15 mg cinnamon oil, the C_max_ and the AUC for 2-methoxy cinnamic acid (0.01 % in essential oil of cinnamon ramulus) were greater than those of cinnamaldehyde (83.49 % in essential oil of cinnamoni ramulus) implying that the active metabolite of the essential oil might be 2-methoxy cinnamic acid [22]. Chen demonstrated that the bioavailability of cinnamic acid was higher when cinnamoni ramulus was fed to rats in contrast to when pure cinnamic acid was fed. Pure cinnamic acid was quickly transformed into hippuric acid. Cinnamldehyde in the cinnamoni ramulus is metabolized into cinnamic acid in the liver [23].

In another study, Anderson et al demonstrated the active ingredient responsible for the anti-diabetic effect of Cinnamon is probably a water soluble constituent. In this study, specimens from C. cassia, C. verum, C, burmanni and C. loureinii were tested. Insulin enhancing biological activity of cinnamon was measured using epididymal fat cell assay. Adipocytes and glucose were incubated in the presence of insulin and or insulin and aqueous extract of cinnamon.

Water-soluble polyphenol polymers from cinnamon, that increase insulin-dependent in vitro glucose metabolism roughly 20-fold and display antioxidant activity were isolated by them and was characterized by nuclear magnetic resonance and mass spectroscopy. The polymers were composed of monomeric units with a molecular mass of 288 [24]. These compounds have been identified as trimers and tetramers of the flavonoids, catechin and epicatechin. These cinnamon polyphenols (CP) with doubly linked procyanidin type-A polymers appear to be unique for their insulin-like activity, because other cinnamon compounds display little or no such activity [24].

The other compounds of cinnamon that showed little or no insulin like activity included; cinnamic acid, cinnamide, cinnamyl alcohol, eugenol and 2-methoxy-cinnamaldehyde under the assay conditions of this study. There was no difference in the insulin like activity between the different species tested.

The available evidence regarding the active compound(s) of cinnamon remains inconclusive. Multiple active compounds at different levels in the insulin signaling pathway is probably a safe intermediary interpretation until more robust evidence emerge.

### Potential mechanisms of action

The mechanism by which cinnamon exerts its anti-diabetic activity is debated and is probably the result of its action at different levels of the insulin-signaling pathway.

#### Insulin receptor

The cellular response to insulin is mediated through the insulin receptor, which is a tetrameric protein consisting of two identical extracellular α-subunits that bind insulin as well as two identical transmembrane β-subunits that have intracellular tyrosine kinase activity [25]. When insulin binds to the α-subunit of the receptor, the β-subunit tyrosine kinase is activated, resulting in autophosphorylation of β-subunit tyrosine residues [26].

Increased autophosphorlylation and decreased dephosphorylation [27] of the insulin receptor is one mechanism that increases the insulin sensitivity. Cinnamtannin B1, a proanthocyanidin isolated from the stem bark of Ceylon cinnamon, activates the phosphorylation of the insulin receptor β-subunit on adipocytes as well as other insulin receptors [28].

#### Glucose transporter 4 (GLUT 4)

GLUT4 is the major glucose transporter in skeletal muscle and adipose tissue, which is under control of insulin. It is well established that insulin promotes the translocation of GLUT-4 from the intracellular compartment to the cell membrane [29]. In diabetes mellitus because of the absence or insufficient sensitivity to insulin, GLUT 4, is decreased. Nikzamir and colleagues reported a significant increase in the expression of GLUT 4 receptor and its mRNA, in cinnamaldehyde treated C2C12 skeletal muscle cells using Real Time PCR [30].

Recently Shen et al reported that cinnamon extract ameliorates type 2 diabetes by inducing GLUT4 translocation *via* the AMPK signaling pathway. The activation of AMPK induces the translocation of GLUT4 to the plasma membrane, and several studies have demonstrated that AMPK and its signaling pathway are potential molecular targets in the development of drugs for the treatment of type 2 diabetes and obesity [19].

Cinnamon increases the amount of GLUT4 receptors as well as Insulin Receptor (IR) and Insulin Receptor substrates [19, 31, 32], thereby facilitating glucose entry into cells.

Shen et al demonstrated that extracts of *Cinnamomum zeylanicum* increased the production and translocation to the plasma membrane of the GLUT 4 in brown adipose tissue and muscle in a dose dependent manner [33]. Anand et al demonstrated a similar finding but quantified the effect by demonstrating an increase of the membrane translocation of GLUT4 from 42.8 % to 73.1 % in cinnamon treated rats when compared to healthy controls [34].

#### Glucose transporter-1 (GLUT 1)

Glucose transporter-1 (GLUT-1) is a receptor expressed on mammalian cells that is responsible for basal glucose uptake into cells. The exact mechanism of activation of this receptor is poorly understood [35]. L 929 fibroblasts are totally dependent on GLUT-1 receptors for glucose uptake into cells [36]. Plaisier and colleagues demonstrated cinnamldehyde increases the GLUT 1 mediated glucose uptake in a dose dependent manner in the L 929 fibroblasts. However in the presence of glucose deprivation in the medium, cinnamldehyde reduced the GLUT 1 mediated glucose uptake [37].

#### Glucagon-like peptide-1 (GLP-1)

Plexopathy demonstrated a dose dependent reduction of serum insulin concentrations and an increase in glucagon-like peptide 1(GLP-1) with cinnamon treatment [38]. Similarly Hlebowicz et al in 2009 reported that the addition of 3 g of cinnamon to a rice meal caused significant increase of GLP-1 levels with decreased serum insulin [39]. Improved glucose transport across the cell membrane reduces the insulin resistance and this probably accounts for the reduced insulin levels.

### Peroxisome proliferator activator receptor (PPAR)

Peroxisome proliferator-activated receptor gamma (PPAR-γ or PPARG), is a type II nuclear receptor that, in humans is encoded by the PPARG gene. This gene encodes a member of the peroxisome proliferator-activated receptor (PPAR) subfamily of nuclear receptors [40]. PPARs form heterodimers with retinoid X receptors (RXRs) and these heterodimers regulate transcription of various genes. Three subtypes of PPARs are known: PPAR-α, PPAR-δ, and PPAR-γ. The protein encoded by this gene is PPAR-γ and is a regulator of adipocyte differentiation. Activation of PPAR lowers plasma triglycerides and elevates plasma HDL cholesterol levels [41], at the same time increasing insulin sensitivity leading to its anti-diabetic effects [42]. PPAR agonists such as Pioglitazone are currently being used as potent anti-diabetic agents in conventional medicine [43].

Cinnamon causes an increase in the expression of PPAR (α) and PPAR (γ), thereby increasing insulin sensitivity [44]. Sheng et al demonstrated that the water-soluble extract of Cinnamon has the ability to induce the expression of PPARα and PPARγ both in vitro and in vivo in mouse adipose tissue. Further, treatment of mouse 3 T3-L1 pre-adipocytes with cinnamon extracts caused them to differentiate into adipocytes [44].

### α glucosidase activity

Adisakwattana studied the inhibitory effects of 4 types of cinnamon on intestinal maltase and sucrase, pancreatic α-amylase, and their combined effect in the presence with acarbose [45].

The HPLC fingerprints of each cinnamon species were established. Among cinnamon species, Thai cinnamon extract was the most potent inhibitor against the intestinal maltase.

They also demonstrated that Ceylon cinnamon was most the potent inhibitor of pancreatic amylase and intestinal sucrase. However its potency to inhibit these 2 enzymes was less than that of acarbose [45]. However when used together with acarbose the cinnamon extracts produced an additive inhibitory effect against all 3 enzymes.

Ranilla et al also demonstrated that Ceylon cinnamon possessed a high dose dependent inhibition of alpha glucosidase and pancreatic alpha amylase [46].

These results suggest that cinnamon bark extracts may be potentially useful for the control of postprandial glucose in diabetic patients through inhibition of intestinal α-glucosidase and pancreatic α-amylase.

### Effects on gluconeogenesis

**Fig. 1:**
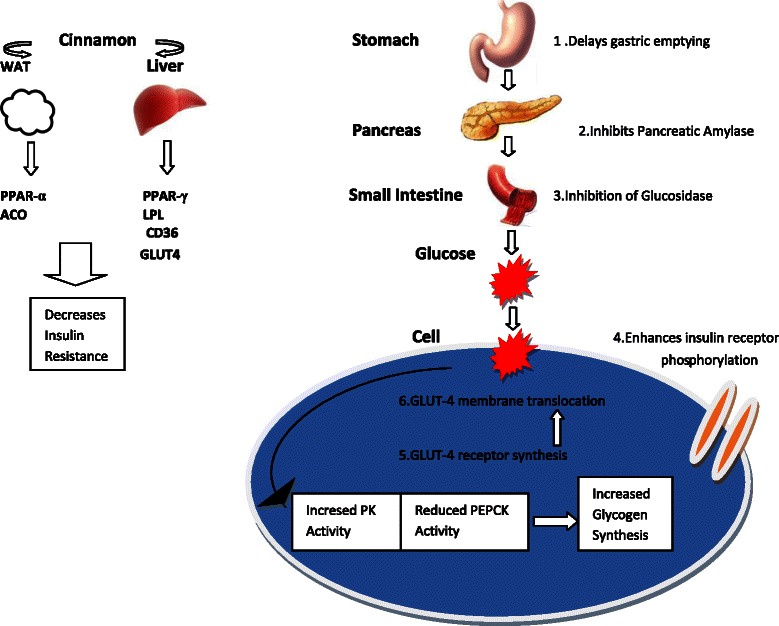
Molecular mechanisms of Cinnamon by which it exerts hypoglycaemic activity. PK: Pyruvate Kinase, PEPCK: Phosphoenol Carboxy Kinase, PPAR-gamma: Peroxisome Proloferator Activated-Receptor gamma, WAT: White Adipose Tissue, ACO: Acyl-CoA Oxidase, GLUT-4: Glucose transporting protein 4, LPL: Lipoprotein lipase, CD36:Fatty Acid Transporter

Pyruvate kinase (PK) and Phosphenol Pyruvate Carboxikinase (PEPCK) are 2 key enzymes regulating the hepatic glucose metabolism. In diabetic rats the PK activity is reduced and PEPCK activity is elevated. The reduced PK activity of Streptozotocin treated rats is restored to near normal values upon treatment with cinnamon. This effect is also observed when diabetic animals are treated with Glibenclamide as well, but the response is less compared to cinnamon [34]. Similarly, significantly elevated PEPCK activity of Streptozotocin induced diabetic rats again nearly normalizes albeit at a slightly higher value than normal values on treatment with cinnamon [34]. The net result is a stimulation of Glycogen synthesis and inhibition of gluconeogenesis, improving glucose metabolism (Fig. 1).

### Gastric emptying

Hlebowicz et al studied the effect of cinnamon on Gastric Emptying Rate (GER) of 14 healthy adults. The GER was measured by using standardized real-time ultrasonography. The subjects were examined after an 8-hour fast if they had normal fasting plasma glucose (FPG) concentrations. The subjects were given 300 g of rice pudding with or without the addition of 6 g of cinnamon. Addition of cinnamon delayed the gastric emptying but caused a more pronounced reduction in post prandial blood glucose and did not affect satiety [47]. However the addition of either 1 or 3 g of cinnamon to a rice meal did not have any effect on the GER or the glucose levels [39].

### Efficacy in animal studies

Ranasinghe et al performed a meta-analysis of studies utilizing *Cinnamon zeylanicum*. In vivo studies utilizing diabetic rats were included, and they analyzed the effect of cinnamon on weight, (FPG), total cholesterol, HDL-cholesterol, triglycerides and serum insulin. Cinnamon was able to check the weight loss of diabetic rats. It also improved the FPG [9].

The result for total cholesterol was conflicting with some trials showing an increase in cholesterol with cinnamon [33, 48] while others showed a reduction [49]. HDL-cholesterol showed an increase with cinnamon treatment [48, 49]. Triglyceride levels also showed a distinct reduction in comparison to controls [9].

Anand et al. demonstrated that the HbA1c of diabetic rats treated with cinnamon and Glibenclamide was less than that of untreated control diabetic rats [34]. Subash Babu demonstrated an HbA1C reduction of 40.2 % in cinnamon treated rats compared to untreated diabetic rats [49]. However, Ranasinghe et al failed to demonstrate a significant fall of HbA1c in cinnamon treated diabetic rats after 30 days treatment, although significant reduction was observed in fasting and postprandial glucose [50]. The failure to demonstrate an improvement in HbA1C may be due to the short duration of 30 days, of this trial.

Kannappan et al. [51] demonstrated the ability of cinnamon to reduce the blood glucose of high fructose diet fed rats. The mean FPG of high-fructose fed rats was high compared to control rats. Treatment with cinnamon caused significant decrease of FPG in the high-fructose fed rats compared to high fructose fed untreated rats. The high fructose fed rats had high serum insulin levels and HbA1c. Upon treatment with cinnamon there was significant decrease of both serum insulin and HbA1c. Additionally the elevated total plasma cholesterol, triglycerides and free fatty acids in the high fructose fed rats normalized following administration of cinnamon.

Shihabudeen et al. studied the effect of cinnamon on post-prandial blood glucose of maltose and sucrose loaded Streptozotocin induced diabetic rats. Cinnamon caused a 65.1 % reduction in the glycaemic response of normal rats following maltose loading. In diabetic rats compared to controls, there was a 78.2 %, 86.3 % and 54.2 % reduction in the glycaemic response when treated with escalating doses of cinnamon of 300 mg/kg, 600 mg/Kg and 5 mg/Kg of Acarbose respectively. There was a similar response with sucrose loading. However the study failed to show a reduction in glycaemic response in glucose treated healthy and diabetic rats treated with cinnamon [52].

Shalaby et al. studied the effect of cinnamon on alloxan induced obese diabetic Sprague Dawley rats. The test animals were initially fed a high fat diet and then injected with alloxan to make them diabetic. Cinnamon administration caused significant reductions in weight, glucose, and lipid levels and normalized elevated liver enzymes while increasing the insulin levels [53].

### Efficacy in human studies

To date, several randomized controlled studies exist that studied the effect of cinnamon on type 2 adult diabetic patients. These studies variably studied the effect of cinnamon on glycosylated haemoglobin, FPG, total cholesterol, LDL cholesterol and triglycerides [54–61].

Seven trials used *Cinnamomum Cassia, Cinnamomum aromaticum* or *cinnamomum burmanii*. 1 trial did not specify the type of cinnamon used [57]. None of the trails used *Cinnamomum zeylanicum.*

The duration of these interventions ranged from 40 days to 4 months, the patient numbers varied from 25 to 137 in each trial and the amount of cinnamon administered per day varied from 500 mg to 6 g.

Of these studies, 2 showed no significant reduction in glucose values [55, 56], while 5 others showed reductions in FPG levels and or glycosylated haemoglobin [54, 57–60].

Khan et al. in 2003 studied a total of 60 patients, who were randomized to receive 1,3, or 6 g of cinnamon daily or a placebo. The background medication consisted of sulphonyureas only. At the end of 40 days, all 3 doses of cinnamon caused a significant decrease in FPG (18-29 %), total cholesterol (12-26 %), triglycerides (23-30 %) and LDL cholesterol (7-27 %) [58]. The change in HDL cholesterol was not significant. This study did not evaluate the effect of the intervention on HbA1c.

Mang et al. randomized 79 type 2 adult diabetes patients on oral hypoglycaemic treatment or diet to receive either 3 g of cinnamon aqueous extract per day or placebo, in a randomized double blind clinical trial of 4 months duration. The study demonstrated a significant decrease of 10.3 % of the initial FPG values. However, the trial failed to demonstrate a significant lowering of HbA1c or plasma lipids [59]. The decrease in plasma FPG correlated significantly with the initial baseline concentrations indicating that patients with a higher initial glucose may benefit more from the addition of cinnamon.

Crawford studied the administration of 1 g of cinnamon as an-add on to usual care of diabetes in 109 type 2 diabetic patients. Participants were randomly allocated to either usual care with management changes by their primary care physician or to usual care with management changes plus 1 g cinnamon capsules daily for 90 days. HbA1c was drawn at baseline and at 90 days and compared with intention-to-treat analysis. Patients receiving cinnamon had a significant reduction of their HbA1c by 0.83 % as opposed to 0.37 % reduction in patients receiving usual care alone [57].

Akilen et al in a randomized, placebo-controlled double blind clinical trial studied the effect of Cinnamon on 58, type 2 adult diabetic patients on oral hypoglycaemics by administering 2 g of cinnamon or placebo daily over a period of 12 weeks. The patients had an HbA1c greater than 7 % at recruitment. The results demonstrated a significant reduction in the HbA1c from 8.22 % to 7.86 % in the treatment group. The study also demonstrated a significant reduction in blood pressure, FPG, body mass index (BMI) and waist circumference at 12 weeks of treatment [60].

Suppapitiporn studied the effect of cinnamon cassia powder in 60 type 2 diabetic patients on oral therapy consisting of Metformin or sulphonylurea in a randomized, placebo-controlled single blind clinical trial. After a 12-week period, HbA1c had decreased from 8.14 % to 7.76 % in the cinnamon group and from 8.06 % to 7.87 % in the placebo group. However the proportion of patients achieving HbA1c < or = 7 % was greater in patients receiving Cinnamon, compared to patients receiving placebo. Nevertheless, it was not found to be statistically significant [54].

More recently, Anderson et al in a RCT randomized 137 diabetic individuals to receive a commercial preparation of cinnamon (CinSulin 500 mg/day) or placebo for 2 months. CinSulin constituted a spray-dried water extract of cinnamon. The study measured the FPG, 2-hr glucose and the insulin resistance (using the HOMA-IR). Additional measurements included the systolic and diastolic blood pressures, serum lipids and fructosamine levels. At the end of 2 months the authors demonstrated a significant decrease of both FPG (In the cinnamon treated group; 8.85 ± 0.36 to 8.19 ± 0.29 mmol/L (*p* < 0.005), compared with 8.57 ± 0.32 to 8.44 ± 0.34 mmol/L in the placebo control group (*p* 1⁄4 0.45), 2-hr glucose values following 75 g glucose load (in the cinnamon extract-supplemented group, 15.09 ± 0.57 to 13.3 ± 0.55 mmol/L (*P* < 0.001), compared with non-significant differences in the placebo group, 14.18 ± 0.60 to 13.74 ± 0.58 mmol/L). Insulin sensitivity, assessed by HOMA-IR, was also significantly improved by cinnamon extract as were fructosamine concentrations [20].

Vanschoonbeek et al studied the effect of 1.5 g of cinnamon per day on insulin sensitivity/glucose tolerance and blood lipids on 25 Type 2 post-menopausal women on oral hypoglycaemic medications over a period of 6 weeks in a placebo controlled study. They demonstrated that cinnamon made no significant improvement in either blood glucose or lipids at the end of the study period [55].

Blevins et al studied the effect of 1 g of cinnamon cassia on blood glucose and lipids in 60 type 2 diabetic subjects in a placebo-controlled study over a 3-month duration. The trial did not show a significant improvement in either blood glucose or lipids at the end of the study [56].

In a systematic review and meta analysis published in 2012, Akilen et al reviewed 6 RCTs (*n* = 435 patients) that studied the glycaemic outcomes of cinnamon in type 2 diabetes. The meta analysis of RCTs showed, a significant decrease in mean HbA1c (0.09 %; 95 % CI was 0.04-0.14) and FPG (0.84 mmol/L; 95 % CI was 0.66-1.02) [62].

Allen et al performed another systematic review and meta analysis of 10 RCTs (*n* = 543 patients) published up to 2012. They demonstrated that cinnamon significantly reduced FPG (-24.59 mg/dl; 95 % -40.52 to -8.67 mg/dl), but not HbA1c levels (-0.16 %; CI -0.39 % to 0.02 %) [63].

A Cochrane review published in 2012 analyzed 10 clinical trials involving 577 patients with either type 1 or type 2 diabetes. The trials used *Cinnamomum cassia* at a mean dose of 2 g per day over a period of 4-16 weeks. The authors concluded that the effect of cinnamon on FPG was inconclusive and there was no significant reduction of HbA1c or post prandial glucose [64].

Ranasinghe et al performed a systematic review and meta analysis of 16 studies that specifically utilized Ceylon cinnamon. However, there were no human trials that utilized Ceylon cinnamon. In animal studies it caused significant reductions of FPG and HbA1c [9].

A summary of the clinical trials is given below (Table 1).

**Table 1:** Summary of clinical trials using cinnamon and their outcomes

		Study design	Duration	Type of cinnamon & dose/day		FPG		HbA_1_C	
						Baseline mean ± SD	Post intervention mean ± SD	Baseline Mean ± SD	Post intervention ± SD
1	Khan et al [58]	Single-blind RCT	40 days intervention 20 days washout	*C.cassia* powder 1 g,3 g,6 g	Placebo	13.77 ± 1.33	13.97 ± 1.23	_	_
					Cinnamon	12.0 ± 1.43	9.1 ± 1.40	_	_
2	Mang et al [72]	Double- blind RCT	40 days	*C.cassia* aqueous extract 3 g	Placebo	8.66 ± 1.47	8.31 ± 1.62	6.71 ± 0.73	6.68 ± 0.7
					Cinnamon	9.26 ± 2.26	8.15 ± 1.65	6.86 ± 1.00	6.83 ± 0.83
3	Suppapitiporn et al [54]	Single blind RCT	12 weeks	C.cassia powder 1.5 g	Placebo	_	_	8.14	7.76
					Cinnamon	_	_	8.06	7.87
4	Vanschoonbe et al [55]	Double-blind RCT	6 weeks	*C.cassia* powder 1.5 g	Placebo	8.28 ± 0.33	8.07 ± 0.36	7.1 ± 0.2	7.2 ± 0.2
					Cinnamon	8.37 ± 0.64	7.91 ± 0.71	7.4 ± 0.3	7.5 ± 0.3
5	Blevins et al [56]	Double-blind RCT	3 months	*C.cassia* powder 1 g	Placebo	8.04	8.02	7.1	7.2
					Cinnamon	7.38	6.84	7.2	7.4
6	Crawford [57]	Randomized placebo controlled trial (no blind/open)	90 days	*C.cassia* powder 1 g	Placebo	_	_	8.28 ± 1.3	7.91 ± 1.5
					Cinnamon	_	_	8.47 ± 1.8	7.64 ± 1.7
7	Akilen et al [60]	Double-blind RCT	12 weeks	*C.cassia* powder 2 g	Placebo	8.77 ± 2.59	8.74 ± 3.11	8.55 ± 1.82	8.68 ± 1.83
					Cinnamon	8.82 ± 3.45	8.04 ± 3.10	8.22 ± 1.16	7.86 ± 1.42
8	Anderson et al [20]	Randomized placebo controlled trial	2 months	C.cassia(CinSulin) 500 mg	Placebo	8.57 ± 0.32	8.44 ± 0.34	_	_
					Cinnamon	8.85 ± 0.36	8.19 ± 0.29	_	_

### Effecacy in pre-diabetes

Roussel et al [65] in a double blind placebo-controlled study investigated the effect of dried aqueous extract of cinnamon on oxidative stress markers and the possible correlation with FPG and plasma insulin levels, in over weight and obese patients with impaired fasting glycaemia over a 12-week period. Twenty-two subjects with FPG in the range of 100-126 mg/dl were selected, and were either given a placebo or 500 mg/d of cinnamon over 12 weeks. In the cinnamon group the impaired fasting glucose levels were significantly reduced (114 ± 2.2 to 102 ± 4.3) at the end of 12 weeks. The reduction in fasting glucose was more pronounced at 12 weeks compared to 6 weeks of supplementation. Similarly, there was significant improvement in plasma oxidative stress markers. However, there was no increase in plasma insulin levels [65].

Similarly, Ziegenfuss in a randomized placebo-controlled double blind clinical trial studied the effect of “ Cinnulin” 500 mg (500 mg of Cinnulin contains 10 g of whole cinnamon powder according to the manufacturer) per day on serum chemistry, body weight, and composition over a 12-week period on 22 adult subjects with the metabolic syndrome and pre-diabetes. At the end of 12 weeks, the subjects in the Cinnulin group had significant decrease of FPG (-8.4 %: from 116 ± 12.8 mg/dl (pre) to 106.5 ± 20.1 mg/dl (post), *P* < 0.01) compared to subjects in the placebo group [66].

### Safety

Anand et al studied the escalating doses of *Cinnamomum zeylanicum* (100 mg/Kg, 200 mg/Kg and 400 mg.Kg respectively), all exceeding therapeutic doses on healthy Wistar rats. They observed no behavioural changes in the animals. Alanine aminotransferase, aspartate aminotransferase, alkaline phosphatase, creatinine and total bilirubin values remained normal during the length of the study period [34].

However animals administered 20 times the effective dose of cinnamon showed a slight, insignificant rise in SGPT, SGOT and ALP, which normalized after 120 hours following administration [34]. This dose is comparable to a human adult dose of 600-2400 mg/Kg.

Subash et al demonstrated that significantly elevated ALP, SGOT and SGPT in streptozotocin induced diabetic rats near-normalized following the administration of Cinnamon [49].

Shen at al studied the effect of cinnamon at variable doses on the glycaemic effect and renal functions of Streptozotocin induced diabetic rats. Animals receiving cinnamon extract in doses exceeding 30 mg/Kg, demonstrated reduction in creatinine values [33].

However, Al-Logmani and Zari demonstrated a significant rise in uric acid and blood urea in streptozotocin induced diabetic rats when compared to controls [67] upon administration of cinnamon.

Thus in animals there is no significant toxicity of cinnamon on the liver, but the results relating to renal functions are controversial, raising the need for more studies to evaluate its effect on the kidney.

A search of the literature for RCTS using human subjects did not reveal any reported significant adverse events when *Cinnamomum cassia* is used in doses of 500 mg-6 g per day. However there were four instances where patients reported rash, hives, nausea and one hypoglycaemic seizure [64].

Although short-term trials have not demonstrated any significant adverse outcomes with cinnamon cassia use, its high coumarin content is a concern during prolonged use [68]. Coumarins are naturally occurring plant compounds with carcinogenic and hepatotoxic properties [69]. Cinnamon cassia which was used in all the human trials, has a higher coumarin content compared to true cinnamon (Ceylon or Sri Lankan Cinnamon) High Performance liquid Chromatography (HPLC) has been used to quantitatively analyze the content. Sproll and colleagues demonstrated the coumarin content of true cinnamon was undetectable while that of cassia cinnamon ranged from 2880-4820mk/Kg using HPLC [70]. Wang and colleagues using a validated Ultra Performance Liquid Chromatography (UPLC) demonstrated the coumarin content of true cinnamon is minimal, amounting to only 0.017 g/Kg, while that of other species of cinnamon varied from 0.31 to 6.97 g/kg [68]. In 1988 the Council of the European Communities set a maximum limit of 2 mg/kg for coumarin in many foods and beverages [68].

This high coumarin content of C. cassia and other species has led some agencies to advocate against the regular use of cassia cinnamon as a supplement in diabetes [71]. On the other hand, the very low content of coumarins found in *Cinnamomum zeylanicum* makes it a potentially useful medication or supplement for long-term use.

### Effective preparations and dose

Different clinical trials have used different preparations of cinnamon. In human subjects, *Cinnamomum cassia* is the only species of cinnamon that has been used and true cinnamon or *Cinnamomum zeylanicum* has not been studied.

All the studies discussed utilized a powdered form of cinnamon except the studies by Mang et al and Crawford which used an aqueous extract of cinnamon and a commercially available preparation of cinnamon respectively [57, 59].

Doses varying from 500 mg to 6 g of cinnamon have been used in studies. However the full dose range seems to be effective and the hypoglycaemic effect is not dose dependent [62].

## Discussion

The in-vivo and in-vitro studies discussed had evidence favoring the ability of cinnamon either in dried powder or aqueous extract forms to improve cellular glucose metabolism. Many mechanisms, such as increased autophosphorylation of the insulin receptor, increasing GLUT-4 receptor sysnthesis and enhancing its membrane translocation, inhibition of pancreatic and intestinal amylase and glucosidase and increasing hepatic glycogen synthesis are probably responsible for this effect.

The studies that described individual mechanisms of action were either in vitro studies or studies that utilized small numbers of experimental animals or healthy human volunteers. The evidence from these studies still remains weak, as the evidence has often been not replicated and some times remained contradictory. Clinical trials have so far not attempted to link the clinical outcomes to presumed mechanism of action. While individual actions of cinnamon are described, the relevance of each mechanism to clinically significant outcomes remains unknown. Future studies should focus upon linking clinical outcomes to mechanistic actions thereby making it more relevant in a physiological context.

The stretpozotocin induced diabetic rat model has demonstrated the ability of cinnamon to normalize glucose metabolism, lipid abnormalities and weight changes associated with diabetes. Most of the trials on animal studies showed the ability of cinnamon to reduce FPG, postprandial glucose and or HbA1c [34, 49, 52]. Cinnamon did not seem to reduce plasma glucose of normal rats compared to diabetic rats [50]. In animal models fed high fructose diets the hyperinsilinaemia, FPG, elevated total cholesterol and triglycerides normalized following treatment with cinnamon [51]. This effectively demonstrates the normalization of the biochemical parameters of an insulin resistant animal model.

However, the clinical trials have proven more difficult to interpret and the results have been more heterogeneous and conflicting.

All studies discussed, had patients with widely varying baseline characteristics such as age, gender and duration since diagnosis; and direct comparison and generalizations may not be valid between studies. There may have been other confounders such as social habits, ethnicity and dietary substances that influenced the action of cinnamon. Although the active ingredient responsible for glucose lowering has not been characterized with certainty, studies have shown that the concentrations cinnamaldehyde vary between species and even among formulations [72]. Consequently it would be difficult to achieve predictable results in clinical trials. Additionally variations in the manufacturing process also affect the quality and availability of the active compound, as these complementary products manufacture is not governed to the same exacting standards of conventional pharmacological products.

Akilen et al and Crawford [57, 60] demonstrated significant reductions in HbA1c with cinnamon use. The baseline HbA1c values for the Crawford and Akilen studies were 8.28 % and 8.55 % respectively. However the Crawford study utilized off the shelf cinnamon capsules from varied sources and the purity and content of cinnamon is not known. A placebo group was not present in this study and the results were compared against a group of patients receiving standard care.

It is interesting to note that the trials that failed to demonstrate a significant decline in HbA1c had a mean baseline HbA1c value close to 7 %. (range of 6.8 % to 7.1 %) [55, 56].

Perhaps a valid explanation for these results would be that the effect of cinnamon is minimal when glucose control is closer to normal and that it exerts a significant effect in reducing glucose as the values increase.

Similarly Khan et al [58] in their study which lasted only 40 days, demonstrated a significant reduction in FPG. The HbA1c was not measured in this study. The baseline FPG was 13 mmol/l and 12 mmol/l in the placebo and treatment groups respectively and the study demonstrated the ability of cinnamon to reduce glucose in the presence of higher baseline values.

However, in contrast, when the effect of cinnamon was studied in patients with impaired fasting glucose, 2 contrasting doses of 10 g and 500 mg of cinnamon per day respectively was able to reduce the FPG significantly [65, 66]. In both these studies, Cinnamon was used as the sole agent that had an impact on blood glucose, in contrast to the RCTS which used cinnamon as an add-on therapy to background hypoglycaemic medications. The background therapy in these studies included oral hypoglycaemic agents prescribed as monotherapy or a combination of medications. This raises the possibility that the glucose lowering potential of cinnamon may have been masked in the RCTS when it was used in conjunction with more potent medications as opposed to being used alone.

The negative outcome of the trial performed by Vanschoonbeek [55] may be attributable to the short duration of 6 weeks, which may have given rise to a false negative HbA1C result. As the life span of red cells is around 120 days, duration of 2-3 months is required to demonstrate a change in HbA1c levels.

A recent Cochrane meta-analysis found that Cinnamon Cassia used in 8 RCTs in 338 patients did not significantly lower the FPG. Similarly there was no significant reduction in HbA1c, in 6 RCTs that studied the effect of Cinnamon on 405, type 2 adult diabetic patients [64].

Hlebowicz demonstrated the potential of cinnamon to delay gastric emptying and concluded that the fall in postprandial plasma glucose in subjects who received cinnamon was disproportionate to the delay induced in gastric emptying [47]. This raises the possibility of a dual mechanism in managing the postprandial glucose surge of diabetic patients; namely the delayed gastric emptying and the inhibition of pancreatic α-amylase and intestinal α-glucosidase.

The safety of long-term supplementation using Cinnamon cassia has been highlighted recently due to its high coumarin content [68]. However, *Cinnamomum zeylanicum*, which only contains trace amounts of coumarins, has not been used in clinical trials. If this form of cinnamon is found to be an effective agent in lowering plasma glucose, the low toxicity figures make it an ideal supplement for long-term use.

Both animal and clinical trials published so far have studied the effect of cinnamon for short durations only. In the clinical trials the maximum length of exposure was recorded by Mang, which lasted for 4 months. Although the adverse events recorded in these studies are minimal, these need to be recorded over longer periods of time with a larger population base.

Some clinical trials have used large doses of cinnamon (6-10 g/day) and the long-term outcomes of such measures need to be studied.

From a safety point of view, there is the potential to use true cinnamon or Ceylon cinnamon for supplementation in view of its low coumarin content. However there are no clinical trials that demonstrate its efficacy, and should be addressed in future clinical trials.

### Integrative medicine and cinnamon

Integrative medicine—an approach that combines conventional and alternative therapies with an emphasis on natural, less invasive evidence-based options—is well suited to the management of complex chronic diseases like type 2 diabetes mellitus [16]. Published research in the field of this new discipline is extremely sparse but many academic institutions offer information, counseling and clinical therapy sessions [73, 74]. Previous work has shown that patients often do not disclose the complementary medicines they use to their physicians but practice these remedies on their own [75–77]. The common reasons for non-disclosure seem to be the passive or negative attitudes towards CAM by conventional physicians and their failure to enquire from patients regarding CAM use [75, 77]. However the focus in integrative medicine would be to allow evidence-based complementary practices alongside conventional therapy, thus empowering the patient to be a contributor of the management team. Conventional health care teams would need to be knowledgeable as well as acknowledge these evidence-based complementary practices. Cinnamon has the potential to be practiced as an integrative medicine in instances of mild diabetes where monotherapy is only required. Similarly it may have a place in instances of high initial FPG values and high HbA1C. Further research on the safety and efficacy of cinnamon as well as the feasibility of it as a component of integrative medicine needs to be performed.

## Conclusion

Both true cinnamon and cassia cinnamon has the potential to lower blood glucose in animal models and humans. Short duration of studies and poor design has made the available evidence difficult to interpret. Ceylon cinnamon has not been studied in humans and its low coumarin content particularly makes it an ideal supplement. Well-designed trials with adequate power to interpret the endpoints are urgently required.

## Abbreviations

CAM: Complementary and alternative medicine
BMI: Body mass index
GLUT4: Glucose transporter type 4
FPG: Fasting plasma glucose
PPAR: Perxisome proliferator-activated receptors
IRSs: Insulin receptor substrates
PI3K: Phosphatidylinositol 3-kinase
PK: Pyruvate kinase
PEPCK: Phosphenol Pyruvate Carboxikinase
